# Supplementary material for: Phaseolus vulgaris Erythroagglutinin (PHA-E)-Positive Ceruloplasmin Acts as a Potential Biomarker in Pancreatic Cancer Diagnosis
Source: Cells. 2022 Aug 8;11(15):2453. doi: 10.3390/cells11152453 (PMC9367852; doi:10.3390/cells11152453)
Supplement: Supplementary file 1 [file cells-11-02453-s001.zip › cells-1775664-Figure S1.pdf]

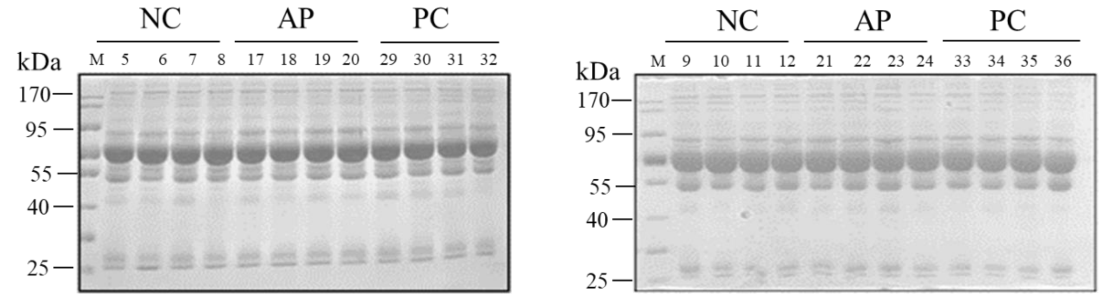

Figure S1. CBB staining of serum proteins from healthy individuals (NC, 5-12), acute pancreatitis (AP, 17-24) and pancreatic cancer (PC, 29-36) patients.
