# Supplementary material for: Phaseolus vulgaris Erythroagglutinin (PHA-E)-Positive Ceruloplasmin Acts as a Potential Biomarker in Pancreatic Cancer Diagnosis
Source: Cells. 2022 Aug 8;11(15):2453. doi: 10.3390/cells11152453 (PMC9367852; doi:10.3390/cells11152453)
Supplement: Supplementary file 1 [file cells-11-02453-s001.zip › cells-1775664-Table S1.pdf]

Table S1. Patients' information.

Group	No.	Gender	Age	Lipase (U/L)	Amylase (U/L)	CA19-9 (U/mL)	Tumor location	Tumor stage	Histopathological classification	Surgery	Metastasis
**Figure 1**											
**NC**											
	1	F*	42								
	2	M*	45								
	3	M*	47								
	4	M*	55								
	5	M*	56								
	6	F*	57								
	7	M*	35								
	8	F*	47								
**AP**											
	9	M*	35	1866	567						
	10	M*	70	4022	937						
	11	F*	30	5341	1971						
	12	F*	64	3971	2050						
	13	F*	58	5277	2288						
	14	F*	60	1252	292						
	15	M*	44	936	248						
	16	M*	46	1796	377						
**PC**											
	17	F*	47			>1000	Head of pancreas	4	-	No	Liver
	18	M*	51			>1000	Head of pancreas	4	-	No	No

	19	M*	51			>1000	Head of pancreas	4	-	No	No
	20	F*	54			>1000	Head of pancreas	4	-	No	Liver
	21	M*	56			>1000	Head of pancreas	4	low grade adenocarcinoma	Yes	Liver
	22	M*	61			>1000	Tail of pancreas	4	-	No	Liver, spleen
	23	F*	67			421.1	Head of pancreas	4	-	No	Liver
	24	M*	79			>1000	Head of pancreas	3	-	No	No
**Figure 4 & Figure S1**											
**NC**											
	1	M	44								
	2	M	47								
	3	F	46								
	4	M	53								
	5	M	54								
	6	M	75								
	7	F	53								
	8	M	45								
	9	M	59								
	10	F	72								
	11	M	61								
	12	M	60								
**AP**											
	13	M	74	1146	148						

	14	F	58	1728	166						
	15	M	63	2191	332						
	16	F	81	1294	1015						
	17	M	36	1815	235						
	18	M	85	3145	867						
	19	M	46	1796	447						
	20	M	66	617	173						
	21	F	42	719	213						
	22	F	16	803	131						
	23	M	79	788	330						
	24	F	86	762	182						
**PC**											
	25	F	61			798.2	Head of pancreas	2	-	No	No
	26	M	71			>1000	Head of pancreas	4	-	No	No
	27	M	69			>1000	-	4	High grade pancreatic ductal adenocarcinoma	Yes	No
	28	F	80			333.6	Body of pancreas	4	-	No	Ovarium
	29	M	51			69.4	Body and tail of pancreas	2	Low grade ductal adenocarcinoma	Yes	Lymph node
	30	M	71			>1000	Body and tail of pancreas	4	Low grade ductal adenocarcinoma	Yes	Liver
	31	M	63			3613.37	Head of pancreas	2	Medium/Low grade ductal adenocarcinoma	Yes	Lymph node
	32	M	58			3839.05	-	4	Low grade ductal	Yes	Liver,

						adenocarcinoma		peritoneum
33	M	48	492.99	Tail of pancreas	4	adenocarcinoma	Yes	Peritoneum
34	F	78	68955.76	-	4	adenocarcinoma	Yes	Liver, lung
35	F	72	67.66	-	3	Low grade adenocarcinoma	Yes	Lymph node Liver
36	M	70	177.6	-	4	Medium/Low grade ductal adenocarcinoma	Yes	Liver

F: female; M, male; NC, normal healthy individuals; AP, acute pancreatitis patients; PC, pancreatic cancer patients; * used for sera pooling
