# Supplementary material for: Phaseolus vulgaris Erythroagglutinin (PHA-E)-Positive Ceruloplasmin Acts as a Potential Biomarker in Pancreatic Cancer Diagnosis
Source: Cells. 2022 Aug 8;11(15):2453. doi: 10.3390/cells11152453 (PMC9367852; doi:10.3390/cells11152453)
Supplement: Supplementary file 1 [file cells-11-02453-s001.zip › cells-1775664-Table S2.pdf]

Table S2. Lectins and their primary sugar specificity

Lectin	Abbreviations	Source	Primary sugar specificity
Aleuria aurantia lectin	AAL	Aleuria Aurantia	Broad specificity to fucosylated glycans
Lens culinaris agglutinin	LCA	Lens culinaris	Fuca1-6 GlcNAc, α-Man, α-Glc
Sambucus nigra agglutinin	SNA	Elderberry bark	Siaα2−6Gal/GalNAc
Phaseolus vulgaris Erythroagglutinin	PHA-E	Phaseolus vulgaris	Bisecting GlcNAc, biantennary complex-type *N*-glycan with outer Gal
Phaseolus vulgaris Leucoagglutinin	PHA-L	Phaseolus vulgaris	Tri/tetra-antennary complex-type *N*-glycan
