# Supplementary material for: Phaseolus vulgaris Erythroagglutinin (PHA-E)-Positive Ceruloplasmin Acts as a Potential Biomarker in Pancreatic Cancer Diagnosis
Source: Cells. 2022 Aug 8;11(15):2453. doi: 10.3390/cells11152453 (PMC9367852; doi:10.3390/cells11152453)
Supplement: Supplementary file 1 [file cells-11-02453-s001.zip › cells-1775664-Table S3.pdf]

Table S3. The values of relative intensity in Figure 1-4.

		NC	AP	PC
**Figure 1a**				
	AAL	28.21 ± 4.16	36.82 ± 9.79	56.96 ± 9.45
	LCA	46.12 ± 4.52	55.42 ± 7.78	66.69 ± 11.58
	PHA-E	49.04 ± 6.92	67.15 ± 11.13	81.80 ± 11.90
	PHA-L	40.72 ± 7.85	51.88 ± 6.25	63.44 ± 6.69
	SNA	43.18 ± 10.71	55.63 ± 11.66	86.22 ± 12.15
**Figure 1b**				
	AAL	35.58 ± 3.57	39.35 ± 1.04	45.18 ± 1.54
	LCA	51.77 ± 2.91	59.57 ± 2.39	68.45 ± 0.80
	PHA-E	32.27 ± 0.83	33.68 ± 1.24	38.59 ± 1.63
	PHA-L	21.84 ± 0.70	28.83 ± 0.12	32.12 ± 1.55
	SNA	53.44 ± 4.79	71.74 ± 7.62	87.71 ± 5.57
**Figure 2c**				
	Bis	2.07 ± 0.12	2.64 ± 0.15	3.14 ± 0.13
	Man	2.90 ± 0.14	3.79 ± 0.24	3.40 ± 0.10
	Fuc	30.24 ± 2.11	33.49 ± 0.78	36.77 ± 0.61
	Neu	73.37 ± 2.11	70.37 ± 0.90	66.93 ± 0.23
**Figure 3b**				
	Cp	19.26 ± 3.06	59.33 ± 7.55	87.33 ± 8.49
	Tf	9.91 ± 1.83	21.38 ± 2.97	25.38 ± 4.75
	Apo-E	8.71 ± 1.64	20.93 ± 2.84	46.83 ± 3.33
**Figure 4b**				
	Cp	10.64 ± 2.25	18.01 ± 4.02	16.87 ± 3.14
	Apo-E	4.45 ± 3.16	16.68 ± 5.75	17.88 ± 7.04
**Figure 4d**				
	Cp	18.72 ± 8.72	37.64 ± 13.65	50.99 ± 17.63
	Apo-E	1.81 ± 1.45	24.25 ± 18.65	17.38 ± 10.32
